# Malaysian Females With Congenital Adrenal Hyperplasia: Surgical Outcomes and Attitudes

**DOI:** 10.3389/fped.2019.00144

**Published:** 2019-04-17

**Authors:** Ani Amelia Zainuddin, Sonia Regina Grover, Chong Hong Soon, Abdul Ghani Nur Azurah, Zaleha Abdullah Mahdy, Loo Ling Wu, Rahmah Rasat, Fatimah Harun, Wee Yan Chia, Khadijah Shamsuddin

**Affiliations:** ^1^Department of Obstetrics and Gynecology, UKM Medical Center, The National University of Malaysia, Cheras, Malaysia; ^2^Department of Pediatric Adolescent Gynecology, Royal Children's Hospital Melbourne, University of Melbourne, Melbourne, VIC, Australia; ^3^Department of Pediatrics, UKM Medical Centre, The National University of Malaysia, Cheras, Malaysia; ^4^Department of Pediatrics, Faculty of Medicine, Universiti Malaya, Kuala Lumpur, Malaysia; ^5^Pediatric Surgery, Department of Surgery, Kuala Lumpur Hospital, Pediatric Institute, Kuala Lumpur, Malaysia; ^6^Department of Public Health, UKM Medical Center, The National University of Malaysia, Cheras, Malaysia

**Keywords:** feminizing surgery, congenital adrenal hyperplasia, external genitalia, ambiguous genitalia, atypical genitalia, genitoplasty

## Abstract

**Background:** Girls born with congenital adrenal hyperplasia have virilized external genitalia. There is considerable debate regarding both the outcomes of feminizing genitoplasty and timing of the surgery in this population.

**Objective:** To investigate outcomes of females 46,XX individuals with CAH in Malaysia, the surgical outcomes of feminizing genitoplasty (FG) and their attitudes toward surgery.

**Study Design:** This is a cross-sectional study involving the two main tertiary centers in Malaysia. All 46,XX patients with CAH and raised female, who had undergone FG were identified and invited to participate. Data on socio-demographic, medical profiles, and attitudes toward surgery were collected. A standardized evaluation of the external genitalia was undertaken including the anatomic and cosmetic evaluation by independent gynecologists.

**Results:** Of 61 individuals identified, 59 participated—consisting of children (*n* = 12), adolescents (*n* = 29) and adults (*n* = 18). All but one had classical CAH (98.3%) and had undergone FG (*n* = 55, 93.2%) with surgery mostly undertaken by pediatric surgeons trained in DSD work (*n* = 44, 74.6%). Complications overall were low (20.3%), with repeat surgery rate of 9.1%. External genital examination was performed in 38 participants. Overall 36.8% had absent clitoral glands and 39.5% had a persistent urogenital sinus and in 10.5%, no vaginal orifices were seen. Poor cosmetic outcomes were present in 42.1% with 55.3% recommended for further assessment under general anesthetic. Almost half participants did not venture an opinion on FG, those who did varied from having a positive attitude toward it (18 participants) to 3 opining that it should not be done, or avoided or delayed. From the participants, 35.5% preferred FG to be done early in life compared to 44.0% of the parents.

**Conclusions:** The reoperation rates of the feminizing genitoplasty surgeries were low however due to the anatomic and cosmetic outcomes, reassessment of the external genitalia of these CAH patients may be required once they consider becoming sexually active as they may require further treatment. Many factors such as cultural sensitivities and access to medical treatment and late diagnoses have an impact on attitudes toward FG.

## Introduction

Congenital adrenal hyperplasia (CAH), is the commonest cause of atypical genitalia in female newborns and is due to excess production of adrenal androgens during fetal development. It is an autosomal recessive condition, with over 90% caused by 21-hydroxylase deficiency (21- OHD) (1). With the development of hormonal treatments, improved neonatal and pediatric care individuals with CAH patients now live into adulthood. Of 46,XX individuals with CAH raised female, surgery for their virilized genitalia has been undertaken in childhood, although in the last decade this has been challenged and in some parts of the world, changing. There is increasing worldwide interest and debate regarding the outcomes of these individuals with reports on various aspects of long-term outcomes (2). To date there has been only one publication on individuals with CAH from Malaysia (3) describing a pediatric population in a government hospital.

Reported outcomes around the world vary significantly and may reflect a number of local issues (4–6). There is a need for more research and study on outcomes of CAH patients in Malaysia and other parts of the world to inform and guide management in each center.

Malaysia is a rapidly developing country in Southeast Asia, with a multi-ethnic Asian, conservative and predominantly Muslim population. This paper will report on the care of 46,XX individuals with CAH, who were raised female in Malaysia. This study investigated the sociodemographic and medical profiles as well as range of other outcomes. This paper will focus on the feminizing genital surgeries or feminizing genitoplasties (FG) performed, the cosmetic and anatomic outcomes and the attitudes of the young women and their families toward such surgery.

This data is clearly important for local pediatric surgeons, patients and their parents to make informed decisions regarding feminizing surgeries.

## Materials and Methods

### Study Design

Participants in this cross-sectional study were 46,XX individuals with confirmed diagnoses of 21-OHD CAH, classical or non-classical, aged >10 years and raised female. Databases from two pediatric endocrine clinics in Malaysia [University Kebangsaan Malaysia Medical Center (UKMMC) and University Malaya Medical Center (UMMC)] as well as the Pediatric Adolescent Gynecology (PAG) unit in UKMMC were searched to identify potential participants. Those who attended for follow up during the 6 month study period and fulfilled the selection criteria were invited to participate.

Study recruitment took place between March 2012 to September 2012. Potential participants were informed about the study by their usual clinicians prior to being approached by researchers. Informed consent was obtained from those who agreed to participate. Where the participant was <18 years old, the parent or legal guardian gave the consent although verbal consent was also obtained from the young person.

Participants were stratified into three age groups: children-group I (aged 10–12 years); adolescents-group II (aged 13–17 years); and adults-group III (aged >18 years).

### Data Collection

This study involved semi-structured interviews using a data collection sheet; review of medical notes and the completion of self-administered translated validated questionnaires which assessed urinary outcomes, recalled gender behavior in childhood, health-related quality of life and body image disturbance questionnaires (the results of which will be reported elsewhere).

The semi-structured interview included data collection regarding socio—demographic details by asking participants and/or their parents. Additionally a number of open questions regarding attitudes to surgery was undertaken. These questions were regarding their feelings and perceptions on feminizing genitoplasty surgery and their perceptions of their healthcare experiences. A brief explanation regarding the different opinions on early vs. late surgery, and single stage vs. two stage surgery was given to participants and their parents prior to asking for their overall opinions on feminizing surgery and on these two aspects of FG based on their own personal experiences. As interviews were not conducted as in-depth interviews and these open questions were to supplement this quantitative study, the answers given were brief and not probed in any depth.

External genitalia examination of the participants was undertaken by the main researcher with a chaperone in the outpatient setting after obtaining separate informed consent from adults participants, or from the legal guardians/parents of younger participants together with their assent to the specific procedure. With separate informed consents, photos with no identification markers were taken. The main researcher is an experienced gynecologist not involved in the management of these participants prior to this study.

A standardized genital examination (4) was used to assess the external genitalia of participants. This included systematic evaluation of genital proportions and symmetry, clitoral size and position, vaginal introitus, labial appearance, and proportions (normal, scrotalized, partial fusion to total fusion), pubic hair distribution and genital skin quality (scarring, pigmentation, and rugosity) (4) with assessment of each aspect separately. Cosmetic outcomes were assigned to categories of “good” (genital appearance normal, unlikely to be judged abnormal by a non-medically trained person), “satisfactory” (up to 2 minor abnormalities, unlikely to be judged abnormal by a non-medically trained person) or “poor” outcome (genitalia appear abnormal; 3 or more abnormal features) (4).

A non-medically trained person is a person who has had no formal medical training, that is, neither a doctor nor a nurse. Examinations were performed in the outpatient clinic, and thus without general anesthetic. Participants had the opportunity to hold a hand held mirror at the time of their examination to enable them to look at their own external genitalia to facilitate their self-education and to involve them in the examination process. Photos of the external genitalia were taken, and a second gynecologist (also not involved in the prior management of these participants) independently scored them. Results were then combined and averaged. Agreement between the 2 gynecologists was made for the data.

The study was approved by research committees of the respective centers. Funding was received from the UKMMC Fundamental Research Fund and the Center for Research and Instrumentation Management (CRIM).

### Statistical Analyses

The continuous variables to describe participants included mean ± standard deviation (SD), range and median (Md); interquartile ranges (IQR) was used for data that was not normally distributed. Frequencies and percentages were used for categorical variables. The normality of data distribution was determined by Kolmogorov-Smirnov tests and by reviewing histograms before performing analyses.

## Results

### Sociodemographic Profiles

Of 61 individuals identified, 59 women were recruited with a mean age of 16.3 ± 4.24 years (range 10–28 years, median 16.0 years, IQR of 6.0 years). There were 12 children, aged 10–12 years (group I); 29 adolescents, aged 13–17 years (group II); and 18 adults aged >18 years (group III). Table 1 shows the socio-demographic profile including ethnicity (64.4% Malays, the main ethnic group in Malaysia), the majority were Muslim (Islam is the main religion in Malaysia and all Malays are Muslims). Four participants did not complete their secondary school education due to bullying at school with three of these four also having learning disabilities. Only one participant was married, only two were sexually active.

**Table 1:** Socio-demographic profiles of all CAH participants and by age group.

**Variables**	**CAH participants**							
	**Age group I 10–12 years (*n* = 12)**		**Age group II 13–17 years (*n* = 29**)		**Age group III** **≥ 18 years (*n* = 18)**		**Total *N* = 59**	
	**f**	**%**	**f**	**%**	**f**	**%**	**f**	**%**
**ETHNICITY**								
Malay	9	75.0	19	65.5	10	55.6	38	64.4
Chinese	0	–	7	24.1	7	38.9	14	23.7
Indian	3	25.0	3	10.3	1	5.6	7	11.9
**RELIGION**								
Muslim	9	75.0	19	65.5	10	55.6	38	64.4
Others	3	25.0	10	34.5	8	44.4	21	35.6
**PRESENT EDUCATION**								
Primary	12	100.0	6	20.7	0	–	18	30.5
Secondary	0	–	20	68.9	8	44.4	28	47.5
Tertiary	0	–	2	6.9	7	38.9	9	15.3
Not completed	0	–	1	3.4	3	16.7	4	6.8
**OCCUPATION**								
Student	12	100.0	28	96.6	4	22.2	44	74.6
Employed	0	–	1	3.4	11	61.1	12	20.3
Non-employed	0	–	0	–	3	16.7	3	5.1
**AVERAGE FAMILY INCOME/MONTHLY (RM)**								
<2000	6	50.0	10	34.5	7	38.9	23	39.0
>2000	6	50.0	19	65.5	11	61.1	36	61.0

### Medical Profiles

Of participants, all but one had classical CAH (*n* = 58; 98.3%): 40 (67.8%) had Salt-Wasting CAH (SW-CAH) and 18 (30.5%) had Simple–Virilizing CAH (SV-CAH). The remaining individual had non-classical CAH (NC-CAH). The SW-CAH variants were diagnosed early in life (range: newborn-6 months) due to atypical genital appearance.

Of the SV-CAH participants, despite all having some degree of virilized genitalia, the age to diagnosis was from newborn up to 13 years of age. The sole NC-CAH participant was diagnosed at age 11 years after investigation for virilization, when she developed acne, a muscular build, and axillary and pubic hair at age 11 years.

The CAH participants were on either prednisolone (*n* = 34; 57.6%) or hydrocortisone (*n* = 25; 42.2%) as their glucocorticoid therapy, and the majority were on mineralocorticoids (*n* = 50; 84.7%), either Florinef or 9-Fludrocortisone. A few (*n* = 8; 13.6%) were on other medications including amlodipine, decapeptyl, metformin, combined oral contraceptives to regulate their menstrual cycles and L-Thyroxine.

All CAH participants except for the one with NC-CAH had virilized genitalia. Almost a third of the CAH participants (*n* = 17; 28.8%) had other illnesses apart from CAH, including epilepsy, PCOS, hypertension, congenital hypothyroidism and asthma.

### Feminizing Genitoplasties (FG)

Four (6.8%) participants have not undergone feminizing surgery (Table 2), including three with classical CAH (aged 12, 12, and 15 years at the time of writing this report) who in infancy were noted to have only mild genital virilisation, and the one with NC-CAH who does not have any genital virilisation.

**Table 2:** Details of feminizing genitoplasty (FG) of CAH participants and by age group.

**Variables**	**CAH participants**							
	**Age group I 10–12 years (*n* = 10)**		**Age group II 13–17 years (*n* = 28)**		**Age group III** **≥ 18 years (*n* = 17)**		**Total *N* = 55**	
	**f**	**%**	**f**	**%**	**f**	**%**	**f**	**%**
**AGE OF RESPONDENT AT FIRST FEMINIZING SURGERY (YEARS)**								
Median (IQR)	2.5	(3.9)	3.0	(2.0)	3.0	(2.0)	3.0	(2.0)
Range	2 months to 6 years		1–6 years		2–13 years		2 months to 13 years	
**COMPLETE DATA ON FEMINIZING GENITOPLASTY (FG) ACCESSIBLE**								
Yes	7	(70.0)	13	(46.4)	5	(29.4)	25	(45.5)
No	3	(30.0)	15	(53.6)	12	(70.6)	30	(54.5)
**PLANNED TIMING OF FEMINIZING SURGERIES PERFORMED**								
Clitoroplasty only	3	(30.0)	16	(57.1)	8	(47.1)	27	(49.1)
Single-stage	7	(70.0)	10	(35.7)	9	(52.9)	26	(47.3)
Two-staged	0	–	2	(7.2)	0	–	2	(3.6)
**POSTOPERATIVE CONSEQUENCES**								
Underwent repeat component of FG	0	3	(10.7)	2	(11.8)	5	(9.1)	
**COMPLICATIONS/PROBLEMS**								
Yes	1	(10.0)	6	(21.4)	5	(29.4)	12	(21.8)
No	9	(90.0)	22	(78.6)	11	(64.7)	42	(76.4)
No information	0	–	0	–	1	(5.9)	1	(1.8)
**PEDIATRIC SURGEON TRAINED IN DSD WORK PERFORMED FIRST SURGERY FOR ALL CAH PARTICIPANTS**								
Yes	9	(75.0)	22	(78.9)	13	(72.2)	44	(74.6)
No	0	–	2	(6.9)	1	(5.6)	3	(5.1)
No information/no surgery	3	(25.0)	5	(17.2)	4	(22.2)	12	(20.3)

The ages at time of first feminizing surgery were not normally distributed. The youngest age group were operated on at a younger age, (median age 2.5 years, range 2–6 months), compared to the older two age groups (median age 3.0 years, range for teenagers 1–6 years, for adults 2–13 years).

Complete data on feminizing surgeries (which include medical records, operating notes and information gathered from interview with parents) were not available for 54.5% (*n* = 30/55) of participants, although information regarding age at surgery, number of operations and complications could be obtained from parents for most. Surgical data was available only from the two main centers (UKMMC and UMMC), despite efforts to obtain notes from other hospitals, this was mostly unavailable for a mix of reasons, including hospital closure and loss of all notes, parents unable to recall surgeons names or dates of surgeries in peripheral hospitals, hence limiting access. Absent surgical data was more common in the older cohorts 15/28 (adolescents) and 12/17 (adult group) compared to 3/10 (children).

Clitoral surgery only was performed in 27 (49.1%) participants with 3, 16, and 8 patients (from Groups I, II, III, respectively). This was the only surgery planned for these participants. A single-stage procedure where all three components of FG (clitoroplasty, labioplasty and vaginoplasty) were planned for infancy or early childhood was undertaken in 26 (44.1%) of all participants, including 7/10, 10/28, and 9/17 for Groups I, II, and III who underwent surgery, respectively. A two-staged procedure where clitoroplasty was done in infancy or early childhood and vaginoplasty was delayed till puberty occurred in 2/28 adolescents.

No major intra-operative or postoperative complications were noted or reported by parents or identified on the available notes. Problems or complications following first FG surgery occurred in 12 participants (20.3%), included vaginal stenosis (*n* = 4), urinary symptoms (*n* = 2), small haematoma (*n* = 1), excessive remaining labial skin (*n* = 5) and scarring (*n* = 2) (several participants had more than one).

Five participants (8.5%) had some form of repeat surgery when they were older. All five had been operated on by different pediatric surgeons for their first procedure, although all surgeons involved had had some training in DSD surgery.

Pediatric surgeons trained in DSD work from the two tertiary centers undertook the surgery on the majority of participants (*n* = 44; 74.6%). Three participants were operated on by surgeons without specific DSD training (a plastic surgeon and the other two were pediatric surgeons), two of the participants were from the adolescent group and the third were from the adult group. For those who had undergone repeat surgery, all except one had been operated the second time by pediatric surgeons with training in feminizing surgeries. The one participant who underwent repeat clitoroplasty and vaginoplasty had the repeat surgery performed by a gynecologist.

### Anatomic and Cosmetic Outcomes of the Feminizing Surgeries

At the time of genital examination only one participant wanted to see her genitalia with a mirror together with the examiner, whilst the others were too embarrassed to do so.

A majority in each age group agreed to be examined with a total of 38 (64.4%) undergoing genital examination (11/12, Group I, 15/29 group II, 12/18 Group III) with the remainder refusing genital examination. Of these, three had not undergone feminizing surgeries. The results (see Table 3) were derived from agreement between both gynecologists involved in the assessment. All 38 participants had genital symmetry. In the majority of participants, the position of the clitori was normal, although almost a quarter in each age group of participants had no visible clitoral glans. A urogenital sinus was present in 15 (39.5%) participants, the vaginal opening was not visualized in four 10.5%) participants and the majority of those examined (*n* = 21 out of 38; 55.3%) had what appeared to be a small introitus. Ten (26.4%) participants had atypical labia majora (with rugosity) and eight (21.1%) had no labia minora.

**Table 3:** Anatomic outcomes of the external genitalia of all the CAH participants (*N* = 38).

**Variables**	**CAH respondents**							
	**Age group I 10–12 years (*n* = 11)**		**Age group II 13–17 years (*n* = 15)**		**Age group III** **≥ 18 years (*n* = 12)**		**Total *N* = 38**	
	**f**	**(%)**	**f**	**(%)**	**f**	**(%)**	**f**	**(%)**
**CLITORIS**								
Absent	3	(27.3)	7	(46.6)	4	(33.3)	14	(36.8)
Small	0	–	4	(26.7)	1	(8.3)	5	(13.2)
Normal	5	(45.4)	3	(20.0)	5	(41.7)	13	(34.2)
Large	2	(18.2)	1	(6.7)	2	(16.7)	5	(13.2)
Excessive	1	(9.1)	0	–	0	–	1	(2.6)
**CLITORAL POSITION**								
Normal	6	(54.5)	7	(46.7)	7	(58.4)	20	(52.6)
Abnormally anterior	1	(9.1)	0	–	0	–	1	(2.7)
Abnormally posterior	1	(9.1)	1	(6.6)	1	(8.3)	3	(7.9)
Absent clitoris	3	(27.3)	7	(46.7)	4	(33.3)	14	(36.8)
**PRESENCE OF UROGENITAL SINUS**								
Absent	5	(45.5)	11	(73.4)	4	(33.3)	20	(52.6)
Present	6	(54.5)	2	(13.3)	7	(58.3)	15	(39.5)
Not visualized	0	–	2	(13.3)	1	(8.4)	3	(7.9)
**VAGINAL INTROITUS**								
Absent	1	(9.1)	1	(6.6)	2	(16.7)	4	(10.5)
Small	6	(54.5)	7	(46.7)	8	(66.7)	21	(55.3)
Normal	4	(36.4)	7	(46.7)	2	(16.7)	13	(34.2)
**VAGINAL INTROITAL POSITION (*n* = 34)**								
Normal	6	(54.5)	11	(73.4)	3	(25.0)	20	(52.6)
Abnormal	4	(36.4)	3	(20.0)	7	(58.3)	14	(36.8)
**LABIA MAJORA**								
Normal	10	(90.9)	10	(66.7)	8	(66.7)	28	(73.6)
Scrotalized or rugated	0	–	5	(33.3)	3	(25.0)	8	(21.1)
Partial fusion	1	(9.1)	0	–	1	(8.3)	2	(5.3)
**LABIA MINORA**								
Normal	9	(81.8)	12	(80.0)	9	(75.0)	30	(78.9)
Absent	2	(18.2)	3	(20.0)	3	(25.0)	8	(21.1)

The cosmetic outcomes, which included genital skin, overall cosmetic outcome (good, satisfactory, poor) and the treatment recommendations from the examiners are shown in Table 4. A good or satisfactory cosmetic outcome was achieved in 57.9%; nine were good (23.7%) and 13 satisfactory (34.2%), respectively.

**Table 4:** Cosmetic outcome and treatment recommendations by type of FG.

**Variables**	**Feminizing genitoplasty (FG) details**							
	**No FG (*n* = 3)**		**Clitoroplasty only (*n* = 18)**		**Planned single stage FG (*n* = 17)**		**Total (*N* = 38)**	
	**f**	**%**	**f**	**%**	**f**	**%**	**f**	**%**
**GENITAL SKIN QUALITY**								
Normal	2	(66.7)	10	(55.6)	7	(41.2)	19	(50.0)
Scarring	0	–	7	(38.9)	9	(52.9)	16	(42.1)
Rugosity	1	(33.3)	1	(5.6)	1	(5.9)	3	(7.9)
**OVERALL COSMETIC APPEARANCE**								
Good	1	(33.3)	6	(33.3)	2	(11.8)	9	(23.7)
Satisfactory	0	–	5	(27.8)	8	(47.1)	13	(34.2)
Poor	2	(66.7)	7	(38.9)	7	(41.2)	16	(42.1)
**TREATMENT RECOMMENDATIONS**								
None needed	0	–	5	(27.8)	2	(11.8)	7	(18.4)
Clitoral surgery	1	(33.3)	1	(5.6)	0	–	2	(5.3)
Vaginal dilatation	0	–	3	(16.7)	0	–	3	(7.9)
Combination	1	(33.3)	0	–	4	(23.5)	5	(13.2)
For reassessment under GA	1	(33.3)	9	(50.0)	11	(64.7)	21	(55.3)

Seven with good cosmetic outcomes are unlikely to need any further surgical interventions and eight (21.1%) have been recommended for vaginal dilatation. Despite a number with clitoromegaly, at this stage no specific surgery is being offered or considered although further discussion initiated by affected individuals may yet occur.

### Attitudes to Surgery and Timing of Surgery

In response to the open questions during the semi-structured interview, many participants and their parents did not give any responses as the questions regarding genital surgery and the timing of this surgery were completely new concepts to them. Only one participant (15 year old who had not had genital surgery) and her mother were aware of these issues as they were fluent in English and had accessed information from CAH websites.

#### Views on Feminizing Surgery

Of individuals who gave responses to the question of feminizing surgery; 18 participants had positive attitude toward feminizing surgery. And further 2 individuals reported that they would have followed whatever the doctors advised and felt it was inevitable, one was adamantly against it, another felt it should be avoided and another thought it may be necessary in the future depending on her future partner. Three participants were reluctant to give an opinion, nine had no particular feelings about it and another six were unaware they had undergone genital surgery. There were 18 who gave no response either because they were too young, had not undergone FG or had learning disabilities and were thus unable to understand the questions.

The views of parents were obtained from the parents who accompanied their children to the clinics thus were mostly parents of the two younger age groups, as the adult participants usually attended clinic alone.

Sixteen parents (with daughters' aged 10–18 years) felt FG was necessary for their children. Reasons given by mothers included: “it was very important that the surgery was done”;. “it was necessary according to their doctors and important for their child's health”; the FG was required as her child's genitalia had looked so “odd.” Reasons given by fathers regarding FG included: “so it is easier to bring her up as the assigned gender”; “if surgery was required and necessary then it should be done as they did not want to have regrets”; “the surgery was necessary for his child for her future to prevent her from feeling “awkward.””

No parents were adamantly against feminizing surgery, however one mother who had refused the surgery when her daughter was an infant felt that the daughter should be involved in the decision-making and would leave it up to the daughter. This was the mother of the 15 year- old who had not had surgery, was fluent in English, was a lawyer and had accessed CAH websites for information. She was able to access information regarding CAH and the controversies surrounding the surgical aspects and decided to delay the surgery until her daughter could be involved in the decision-making processes.

One father felt he could not comment as they had not been involved in the decision as he and his wife had adopted their daughter with CAH only after she had been abandoned by her parents and after she had had the feminizing surgery. Twenty-eight parents did not express any opinion (their daughters were aged 10–22 years) and no opinion was obtained from nine parents as they did not attend the clinics. Three other parents were advised that surgery was not necessary as their daughters had mild virilization or no atypical genitalia (the single non- classical respondent).

#### Views Regarding “Best Age” for Undergoing Feminizing Surgery

The responses from participants and parents to the open question regarding the most appropriate age which they felt FG should be done: infancy, early childhood (ages 2–4 years, in the pre-school period), puberty or adolescence and adulthood are shown in Table 5.

**Table 5:** Participants and parents' age preferences for first feminizing genitoplasty (FG).

**Variables**	**Age of FG in infancy (*n* = 8)**		**Age of FG after infancy (*n* = 47)**		**No FG done (*n* = 4)**		**Total (*N* = 59)**	
	**f**	**%**	**f**	**%**	**F**	**%**	**f**	**%**
**PARTICIPANTS' PREFERRED AGE OF FG**								
Infancy	2	(25.0)	9	(19.1)	0	–	11	(18.6)
Early childhood	2	(25.0)	8	(17.0)	0	–	10	(16.9)
Puberty	0	–	5	(10.6)	0	–	5	(8.5)
Adulthood	0	–	2	(4.3)	1	(25.0)	3	(5.1)
Others	4	(50.0)	23	(48.9)	3	(75.0)	30	(50.9)
**PARENTS' PREFERRED AGE OF FG (FOR THOSE WHO GAVE OPINIONS, *n* = 29)**								
Infancy	4	(50.0)	9	(19.1)	0	–	13	(22.0)
Early childhood	0	–	13	(27.7)	0	–	13	(22.0)
Puberty	0	–	1	(2.1)	2	(50.0)	3	(5.1)
Adulthood	0	–	0	–	0	–	0	–
No opinion given	4	(50.0)	(24.0)	(51.1)	2	(50.0)	30	(50.9)

Of young people, 30 did not choose which age; three were not sure and would follow their parental advice (5.1%); six who were not aware they had undergone FG (10.2%); one did not agree with FG at all (1.7%) and 20 (33.9%) offered no opinion- they were either too young, had learning disabilities or had not undergone FG at all.

Of participants who gave an opinion (*n* = 29), the majority preferred to have their FG done early either in infancy (11/29; 37.9%) or in early childhood (10/29; 34.5%). There were, however, participants who wanted to have their surgery for the first time to be done at puberty (5/29; 17.2%) and even in adulthood (3/29; 10.3%). For those who had the surgery after infancy (*n* = 47), nine (19.1%) preferred for it to be done in infancy, and for those who had FG in infancy (*n* = 8), none who opined preferred it to be done after early childhood.

The participants were also asked to give reasons for their age preferences. The 11 respondents (aged 14–27 years) who preferred FG in infancy gave the following reasons; early surgery would result in less psychological trauma, less physical pain (as babies don't remember) and less embarrassment (“malu” in Malay) when the child grows older. More detailed responses included: “that she would have felt more upset if the surgery had been delayed till adulthood”; “it would be easier for the doctors and parents for the surgery to be done in infancy as babies ‘don't talk much”';. if the individual gets older without having had the surgery, this will be “malu” (embarrassing/shameful) for her and “what will people think?”

For the participants (age 13–22 years) who preferred the feminizing surgeries to be carried out in early childhood, their reasons for this age were; to reduce psychological trauma for both participants and their parents, to be in line with parents' decisions for early childhood surgery, to prevent gender identity issues when they grow older and again, to prevent “malu” (embarrassment).

Five participants (aged 13–24 years) preferred surgery to be done at or just prior to puberty giving the following reasons: it was important that the child understands what is going on, can be involved in the decision-making and “it is not too late” for surgery to be done.

Three participants, aged 14, 15, and 21 years thought feminizing surgery should be delayed till adulthood. Reasons given included: the decision should be delayed to allow the individual to have a say (this person has requested gender reassignment to male); delaying till the surgery is needed—to allow pregnancy to occur; delay till it is established that the FG is required for sexual activity and to allow a husband to be involved in the decision-making for the surgery.

The preferred age of FG was given by 29 parents and the results are also shown in Table 5. The mothers of two participants who had not undergone surgery also gave opinions which are included in the table. None wanted their child to have their surgery in adulthood, and none refused surgery completely. The majority wanted surgery to be done early in their childs' life; either in infancy (22.0%) or early childhood (22.0%). Of parents with children operated on in infancy (*n* = 4), all still preferred their child to have surgery at that age.

When asked to give reasons for their preference, 11 of 13 parents who chose infancy as their preferred age of first FG (with children ages 10–17 years) reasons given included: babies heal faster, less psychological trauma for the children and their parents as babies will not remember the surgeries and parents' anxiety over the genital ambiguity will be reduced, it is easier to handle the child as a baby compared to a toddler or an older child, easier to send the babies for childcare after surgery and less “shame” involved.

One mother with two daughters with CAH preferred first surgery in infancy. In her experience, one daughter had undergone surgery in infancy and the other at age 4. She reported it was far easier to care post-operatively for the infant than the 4 year-old. One father emphasized that although he prefers infancy, he would follow the experts' advice.

For the 13 parents who preferred early childhood to be the age for their children's first FG, 12 gave their reasons. These were: at this age, the child is not yet schooling hence there are no school issues to deal with, at this age it is easier to care for the child post-operatively (the child is not yet embarrassed), easier surgery as parts are smaller and will grow and heal better, to prevent gender-related issues that may occur when the child grows older and less psychological trauma. In fact, three parents specifically stated that if FG were done in early childhood, this will prevent them from embarrassment in their teens.

One father opined that it was better psychologically for both parents and their children, so parents can know for sure which gender to raise their child as. Another father says, “[I prefer the surgery to be done] in early childhood [at the age] of 2–3 years, to settle the child's atypical genitalia early so the child can be like everyone else.”

For parents who felt that the surgery should be delayed (*n* = 3), all felt that the young person should understand and be involved in the decision-making.

With regards to the parents of 30 participants did not give their opinions on this matter, for the majority this was because they did not accompany their children to the clinic hence were not available for the interviews or for those who were, they were reluctant to participate.

## Discussion

There is a scarcity of published data on outcomes of patients with CAH in Malaysia. The sample included is a relatively large group of CAH participants recruited from the two main referral centers for CAH patients in Malaysia. The study population was representative of the different ethnic groups in Malaysia. This study is also likely to represent the majority of women with CAH in Malaysia. Never the less there are likely to be a number who have been lost to follow-up, or who have swapped gender and thus have not been identified.

Newborn screening for CAH has not been introduced in Malaysia yet and there are no national databases. The participation rate in this study is high, although, for largely cultural reasons genital examinations were refused by almost half of the study participants. Likewise the sexual activity rates were low—partly for cultural reasons and for young age.

The sample size in this report is relatively large compared to previous reports on FG outcomes, which have included from 6 to 69 participants (7, 8) with even recent papers in 2016 included samples of only 30 and 37, respectively (9, 10).

The majority of the participants (93.2%) had undergone feminizing surgery with a median age of surgery of 3.0 years. The timing of FG is comparable to other studies reporting first surgery at age 3.0 years (6, 7, 11–14) with these studies coming from both developing (Turkey, Brazil, India) and developed nations (The Netherlands and Germany). Other countries (Australia, United Kingdom, United States of America, Israel, Canada and Italy) and Egypt have however reported their age of FG to be earlier, at <2 years (4, 8, 9, 15–19). For Malaysia, the factors influencing timing of surgery have included both delayed diagnosis and referral to specialized centers; lack of awareness by birth attendants or primary health care givers regarding identification of virilized genitalia and even reluctance in seeking medical treatment by the parents (2). There is a paradigm shift in many countries now where there is a tendency to delay surgery arising out of concerns regarding poor outcomes as well as in regard for patient autonomy in making decisions about genital surgery.

Undertaking examination of the external genitalia was challenging due to this being a source of embarrassment and culturally taboo. Additionally, vaginal examination was not undertaken as the patients were young and not sexually active, thus examinations when undertaken, were limited to visualization with labia spread apart. Examinations under GA were not an option due to inconvenience to the patients and their families, increased likelihood of refusal, additional costs to families and difficulty in justifying this clinically. Thus, the results of the genital examination are of limited accuracy.

The clitoral outcomes (absent, small, large/excessive) is similar to the range reported by others (4, 6, 12, 20, 21). In Malaysia prior to 2006–2007, clitoral preserving surgery was not performed, resulting in a high prevalence of absent clitori (36.8%) amongst the many CAH patients due to clitorodectomies (22). The other abnormal clitori were predominantly in the young women who have not undergone surgery, who had larger clitori.

Previous studies (4, 7, 12, 19–21) report small/strictured/stenosed/inadequate vaginas ranging from 28.1% (19) up to 73.0% (4), whilst this study found 55.3%. However, the accuracy of these findings was limited as it was based on external observation without internal pelvic examinations. Our results may change if vaginal examinations either awake or under GA were performed.

Overall cosmetic outcomes were: good (*n* = 9, 23.7%), satisfactory in 13 (34.2%) and poor in 16 (42.1%). The standardized assessment used in this study had been introduced by Creighton and also used in UK (4), Australia (5) and Canada (17). Among these studies, our study reported poor outcomes of 42.1%, similar to the UK results of 41.0% (4). The Australian study reported only 6% poor outcomes whereas the Canadian study reported good outcomes in 100% (5, 17). The explanations may be different for each site. The UK cohort reflects a potentially biased, selected sample with their study cohort coming in part from a support group and part from a referral center where the complex cases from all over the UK are seen, with no details given with regards to how many surgeons were involved. The Melbourne study reflects a cohort from one center with the majority of surgery done by one surgeon. The Canadian study involved assessment by the operating surgeon which introduces a different sort of bias.

The responses from individuals with CAH and their parents regarding their perception of the role of FG were limited by many not giving or having an opinion. Of those who did respond the majority preferred that the surgery be performed early in life.

There are few studies which ask for patients and parents' perceptions or opinions regarding surgery, the preferred age or the timing for feminizing surgery. The questions, when asked, are not standardized making any comparison challenging. Most of the studies done have reported on outcomes of the FG from the surgeon's point of view with a few asking regarding feedback from the patients on their assessment of the cosmetic outcome of their external genitalia following FG.

The reasons previously given by clinicians who had advocated for early surgery included normalizing the appearance, enabling gender assignment, surgery being technically easier in infants compared to older children and for psychological reasons to allay parental discomfort, decrease the child's memories of traumatic surgery and to ease the transition to adulthood (23).

Those who oppose early genital surgery say there is insufficient evidence that early surgery benefits gender identity, that this causes potential impairment in clitoral sensitivity and results in high rates of revision vaginoplasties (24) and that the individual needs to be involved in the decision making as a matter of human rights. There are no reports at present, as far as the authors are aware, on the psychological benefits of early vs. later surgery in girls with CAH. Never the less there is some information in boys who undergo surgery for correction of severe hypospadias that boys who have completed their hypospadias corrective surgery at a younger age, have better self-image and self-esteem and feel more positive about sexual relationships when assessed as teenagers, compared to boys who completed their surgeries later (25). This is despite no difference in initial hypospadias severity or anatomical and functional outcomes of the surgery. Thus, there may be detrimental impacts of delaying surgery that have yet to be seen in females with CAH whose surgery is delayed.

The challenge of assessing outcomes relating to feminizing genital surgery include the small sample sizes of studies, the variability of cases, the loss to followup over time, the surgical expertise, and techniques used which have changed over time (23). Furthermore, the potential impact of cultural differences may mean that results are not necessarily applicable in different countries with different cultures.

In Malaysia, the surgeon who had operated on the majority of these patients did so with the best of intentions, in line with the recommendations. This is a situation that equally applies to other pediatric surgeons around the world—remembering also that CAH was a condition that resulted in death for many individuals until it was identified. The pediatric surgeon in Malaysia who had been undertaking this work was courageous and inquisitive enough to ask the main researcher to investigate the outcomes of the feminizing surgery. This surgeon has also changed his approach toward performing feminizing surgery to his CAH 46XX patients, being more conservative now and encouraging delay in surgery to allow the young person to be involved in making their own decisions with regards to feminizing surgery.

A further aspect that requires consideration in Malaysia is that with the exception of two or three patients and their parents who could speak English and were able to access the Internet for extra information, the other participants had limited access to further information regarding CAH. The institutions involved in managing these patients in the past did not produce information booklets on CAH and there is no formal support group for CAH. Clinical care is further compounded by limitations in health knowledge in the community. Furthermore, the “malu” factor is prevalent in Malay society with its equivalent in many Asian communities. Thus, most families would not have informed other people about their child's condition and they would be unwilling to join a support group to “share.” With the exception of five families, the others did not know of other families with CAH. Therefore, whatever knowledge or reasons that these participants and their families were given for having early surgery comes solely from the clinicians who managed them early on. “Follow doctors' advice” was repeated several times by the participants and parents. It would never have occurred to them to question the doctor's advice.

The concept of “Malu” is important to understand the impact of culture on perception and decisions. “Malu” in Malay societies means shame, shy, bashful, embarrassed, a concept that has effects in a wide array of personal and social realms including in the construction of gender differences (26). The first lessons in learning shame or to be “malu” begins at an early age and is thus integral to the Malay psyche. For example, when bathing a child, the mother or older person attending to the child may exclaim that it is shameful that their genitals are showing, this shame at exposure of the body is gradually extended to a concern with exposure of the self to strangers, a type of shame that may be described as shyness (26).

In Western societies, shyness is generally considered to be a matter of behavior or temperament and is viewed as a negative feeling (27). However, in Malay societies, “malu” designates appropriate shyness, demonstrating respect for elders and elites (26) and thus by extension medical personnel. The concept of “malu” is also collectively shared, for example knowing “malu” involves a child learning that his or her identity is bound to others, especially family members, as “malu” can be evoked by the actions of a close relative. Thus, having early genital surgery would prevent “malu” not only in the participants but also for the parents. Certainly, shame in having atypical genitalia, and having surgery to “normalize” one's genitalia is not confined to Malaysian society only, with depression, stress, secrecy, stigmas found in many other societies (2, 23, 28, 29).

A study on parental perspectives in a Western nation on medical treatment of intersex reported that of 21 parents of children with 46 XX CAH, when asked to give reasons for their consenting to FG, all the parents cited the potential for a more natural genital appearance, 89% reported the potential for better sexual function and 79% reported improvement in urinary function as the critical determinants (30). When asked if they would consent to genital surgery if a reduction in sexual sensation/responsiveness were certain, 95% of parents indicated they would still consent (30). Most parents in this study indicated that they thought it was their responsibility to make decisions concerning surgery and all disagreed with postponing surgery until the child was old enough to consent.

These findings differ from those of our study, as the reasons given by the parents do not include sexual capability or to alleviate urinary problems and three of our Malaysian parents actually considered delaying surgery to enable the child to be part of the decision-making. A study in Vietnam has also reported that parents of their CAH patients were more likely to be happy with the results of the surgeries if the surgeries had been done earlier (28) and a study has reported that early genital surgery was preferred by individuals with DSD of whom 15 of the 24 had CAH (31).

It may be that some of the differences in the opinion regarding genital surgery may lie with the underlying conditions. The satisfaction with genital surgery amongst individuals with 46 XY DSD has shown a preference that genital surgery should be minimized and performed mainly in adolescence or adulthood with the patients' consent (32). A German study by Brinkmann et al. (33) also examining patients' perspectives of their treatments reported that 15 of 37 participants with different DSD conditions had negative memories when it came to their surgical interventions. These authors noted those with CAH differed in many ways from the participants with XY DSD, especially with regards to timing of interventions, their current psychological well-being and the retrospectively judged satisfaction with aspects of treatment. The CAH patients seem more physically and psychologically stressed from the amount, invasiveness and form of medical treatment than others (33).

In our study, the one CAH participant who very clearly disagreed with all genital surgery is one who wishes to change to a male gender—although this individual represents someone with a delayed diagnosis, delayed surgery and limited medical attention—which is not the typical experience of individuals with 46,XX CAH in developed countries.

Thus, from the participants and parents' perceptions and preferences, bearing in mind that <50% of participants and parents participated in this part of the study, that it would seem that early feminizing surgery is generally preferred It should also be noted that there has been a shift in surgical attitude over the same time, so that less surgery is likely to be performed, particularly in those who are diagnosed late.

## Conclusion

The anatomic and cosmetic findings of the external genitalia of the female CAH patients in this study are overall comparable to the results in other studies, however, due to the limitations of the assessments in the outpatient clinic in young CAH participants of whom the majority were not yet sexually active, there needs to be repeated assessment in the future when the CAH participants are ready to be examined internally once they have been or attempted to be sexually active. They may then require further treatment and care for their sexual issues by gynecologists. The impact of differences in culture and country may also play a role in responses but also attitudes toward genital surgery. Cultural sensitivity which impacts on family attitudes, as well as access to medical care and community health education need to be considered in terms of making decisions regarding surgery.

## Ethics Statement

The study was approved by research committees of the respective centers (UKM Medical Research and Ethics Committee, Approval Reference FF-318-2011 and UMMC Medical Ethics Committee, Approval Reference 859.11).

## Author Contributions

AZ, AN, LW, RR, FH, and WC recruited the patients for the study and collected the data. AZ, ZM, SG, and KS were responsible for the research project design and the analyses of data and in the writing of the final report. AZ, SG, and CS prepared the final draft of the manuscript for publication.

### Conflict of Interest Statement

The authors declare that the research was conducted in the absence of any commercial or financial relationships that could be construed as a potential conflict of interest.
